# The Amino Acid Transporters of the Glutamate/GABA-Glutamine Cycle and Their Impact on Insulin and Glucagon Secretion

**DOI:** 10.3389/fendo.2013.00199

**Published:** 2013-12-31

**Authors:** Monica Jenstad, Farrukh Abbas Chaudhry

**Affiliations:** ^1^Institute for Medical Informatics, Oslo University Hospital, Oslo, Norway; ^2^Centre for Cancer Biomedicine, University of Oslo, Oslo, Norway; ^3^Institute of Basic Medical Sciences, University of Oslo, Oslo, Norway; ^4^The Biotechnology Centre of Oslo, University of Oslo, Oslo, Norway

**Keywords:** GABA, glutamate, glutamine, insulin, SAT2, Slc38a2, Slc38a3, SN1

## Abstract

Intercellular communication is pivotal in optimizing and synchronizing cellular responses to keep homeostasis and to respond adequately to external stimuli. In the central nervous system (CNS), glutamatergic and GABAergic signals are postulated to be dependent on the glutamate/GABA-glutamine cycle for vesicular loading of neurotransmitters, for inactivating the signal and for the replenishment of the neurotransmitters. Islets of Langerhans release the hormones insulin and glucagon, but share similarities with CNS cells in for example transcriptional control of development and differentiation, and chromatin methylation. Interestingly, CNS proteins involved in secretion of the neurotransmitters and emitting their responses as well as the regulation of these processes, are also found in islet cells. Moreover, high levels of glutamate, GABA, and glutamine and their respective vesicular and plasma membrane transporters have been shown in the islet cells and there is emerging support for these amino acids and their transporters playing important roles in the maturation and secretion of insulin and glucagon. In this review, we will discuss the feasibility of recent data in the field in relation to the biophysical properties of the transporters (Slc1, Slc17, Slc32, and Slc38) and physiology of hormone secretion in islets of Langerhans.

## Introduction

The endocrine cells of the pancreas and the cells of nervous system have different functions but also reveal interesting similarities. Islet cells of Langerhans secrete insulin and glucagon and are considered to be necessary for maintaining glucose homeostasis. However, neurons may also release insulin, and compelling evidence have been provided for a brain-centered glucoregulatory system that work in concert with the islet cells to regulate plasma levels of glucose (1–3). Interestingly, recent advances in the field reveal mechanistic similarities in the regulation of insulin and glucagon secretion in the islets as compared to neurotransmitter release. Amino acids, which play pivotal roles in fast neuronal signaling, have also been proposed to act as signaling molecules in the islets of Langerhans (4–7). This is consistent with the detection of significant changes in plasma glutamine concentrations in newly diagnosed diabetic patients (8) indicating that dysfunctional amino acid metabolism, signaling, and/or amino acid transporter function may precede and/or augment development of diabetes. In this review, we will discuss the significance of glutamate, GABA, and glutamine and their transporters in the regulation of insulin and glucagon secretion in the pancreas.

### Amino acid transporters and neuronal signaling

Classical neuronal signaling is a form of paracrine signaling where a neurotransmitter is released at the synapse from a neuron and the message is conveyed by activation of specific receptors on the surface of an adjacent neuron. Several amino acids play fundamental roles in synaptic transmission. Glutamate and GABA are the main fast excitatory and inhibitory neurotransmitters, respectively. After their release, the signal is partly inactivated by transport of the released neurotransmitter into astroglial cells and conversion to glutamine catalyzed by glutamine synthetase (GS) (9, 10). Glutamine may then be released from astroglial processes and shuttled back to neurons for regeneration of the transmitters by the neuronal phosphate-activated glutaminase (PAG) (11, 12). Existence of such a cycle, known as the glutamate/GABA-glutamine (GGG) cycle, is bolstered by the demonstration of glutamate and GABA transporters on synaptic vesicles and on perisynaptic astroglial plasma membranes (13–17), and by the characterization of the Slc38 family of amino acid (glutamine) transporters (18–20). We have demonstrated that the Slc38 family members SN1 (Slc38a3) and SN2 (Slc38a5) release glutamine from the astrocytes (18, 21, 22), while SAT2 (Slc38a2) imports glutamine into glutamatergic neurons and maintains neurotransmitter pools of glutamate involved in retrograde signaling (23). The Slc38 family member SAT1 (Slc38a1) is selectively localized in inhibitory neurons supporting uptake of glutamine for GABA formation (19, 24). Consistent with a role of SAT1 in the GGG cycle, the SAT inhibitor MeAIB reduces GABAergic inhibitory synaptic transmission (25, 26). However, the presence of these transporters also in peripheral organs suggests important roles in cell-specific metabolism and/or in non-neuronal signaling (27–29).

### The endocrine cells of the islets of Langerhans harbor proteins involved in classical neuronal signaling

The endocrine cells of the pancreas share many characteristics with the cells of the central nervous system (CNS). The two tissues share a similar transcription program (30) and have extensive similarities in global mRNA expression and chromatin methylation (31). In addition, transcription factors like Pax6 and Nkx6.1 are important for development and differentiation of both neurons and islet cells (32–34).

The endocrine islet cells also contain most of the components involved in synaptic transmission in the CNS. In addition to the hormone loaded secretory granules (SGs), islet cells contain synaptic-like microvesicles (SLMVs) resembling synaptic vesicles found in nerve terminals (35, 36). The exocytosis of SGs and SLMVs also share many similarities with the exocytosis of synaptic vesicles. As in the CNS, exocytosis is dependent on the opening of voltage-dependent Ca^2+^ channels (37, 38), and on the complex assembly of SNARE proteins including SNAP25 and syntaxin coupled to the vesicle and to the target membrane (39). Furthermore, exocytosis and hormone secretion is in both regulated by myotrophin, and the same miRNA, miR-375, regulates translation of its gene in both tissues (40). In addition, the biogenesis of SLMVs in β-cells is dependent on the same adaptor protein complex as in GABAergic neurons (41).

There is co-release of glucagon and glutamate from α-cells (42), and SLMVs of β-cells contain high levels of glutamate (43). In β-cells, glutamate activation of AMPA and kainate receptors stimulates Ca^2+^ influx and insulin secretion (44, 45), whereas mGluR stimulation, both inhibit and stimulate insulin secretion depending on the concentration of glucose (46, 47). In addition, glutamate stimulates GABA release from SLMVs in β-cells independently of insulin release (48). In α-cells, glutamate inhibits glucagon secretion. Thus, glutamate is involved in both paracrine and autocrine regulation of glucagon and insulin.

When it comes to GABAergic signaling, GABA_A_ receptor activation is important for β-cell autocrine feedback (49), and it reduces glucagon secretion from α-cells (50, 51). Furthermore, the key enzymes involved in the metabolism of the neurotransmitters glutamate and GABA, e.g., PAG, glutamic acid decarboxylase (GAD), and GS, are all present in islet cells (4, 52, 53). In fact, islet β-cells contain GABA and GAD at the same levels as GABAergic neurons (54, 55), and β-cells have the highest GABA concentration outside the CNS.

### Glucose and amino acids in the regulation of insulin secretion

**Figure 1:**
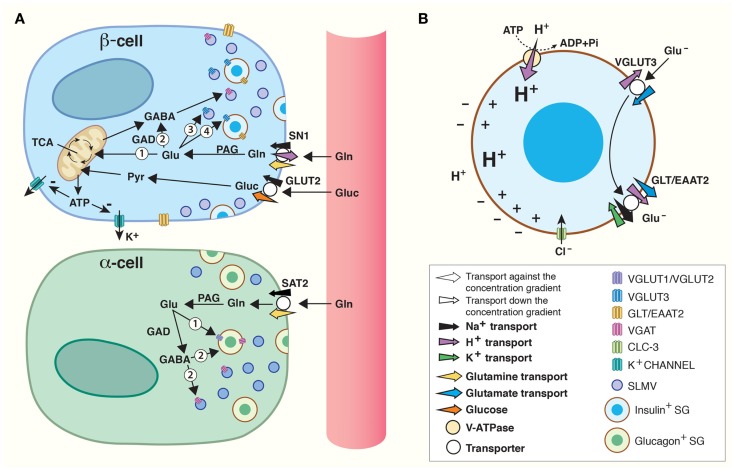
**Amino acids in pancreatic endocrine signaling and insulin maturation**. **(A)** Amino acids are shuttled between cells and intracellular compartments and are involved in autocrine and paracrine signaling in the pancreatic islets of Langerhans. High levels of glucose (Gluc) are carried by the blood flow to the islet β-cells where glucose is accumulated by a glucose transporter (GLUT2). Through glycolysis, glucose is catabolized to pyruvate (Pyr) which is transported into mitochondria for oxidative phosphorylation. The product, ATP, inhibits K^+^-channels. As a result, β-cells are depolarized and this stimulates fusion of insulin containing secretory granules (SGs) with the plasma membrane. Glutamine carried by blood can be accumulated inside β-cells by SN1 on their plasma membrane. Glutamine may be converted to glutamate by phosphate-activated glutaminase (PAG) and enter the TCA cycle inside mitochondria (1) to produce ATP which may regulate K^+^-channels in the same way as glucose. Alternatively, glutamate may be translocated into synaptic-like microvesicles (SLMVs) (3) or insulin containing SG (4). Finally, glutamate may be metabolized to GABA (2) by glutamic acid decarboxylase (GAD) and accumulated inside SLMVs for secretion. In α-cells, SAT2 accumulates high levels of glutamine which may be metabolized to glutamate and/or GABA by PAG and GAD, respectively. The vesicular glutamate transporters (VGLUT) 1 and 2 and VGAT then transport glutamate (1) and GABA (2) into SLMVs and glucagon containing SG for exocytotic release. **(B)** VGLUT3-mediated transport of glutamate into SGs, followed by GLT/EAAT2-mediated transport out, contributes to maturation of insulin. A vacuolar (V)-ATPase generates a high electrochemical gradient for H^+^. Flux of H^+^ through VGLUT3 and down its electrochemical gradient energizes transport of glutamate (Glu^−^) into SGs. Subsequently, the glutamate transporter GLT/EAAT2 translocates glutamate out of the SG, and this transport is coupled to 3 Na^+^ and 1 H^+^ transport in symport and 1 K^+^ in antiport. The concerted action of VGLUT3 and GLT/EAAT2 results in a net movement of positive charge out of the SG. This counteracts inhibition of the V-ATPase and augments accumulation of H^+^ inside SG. Such acidification stimulates conversion of pro-insulin to insulin. The chloride channel CLC-3 also resides on the membranes of SG. Transport of Cl^−^ down its electrochemical gradient (into SG lumen) by CLC-3 also counteracts inhibition of the V-ATPase and increases the luminal acidification which stimulates maturation of insulin.

The main triggering signal for insulin release from β-cells is elevated plasma glucose levels. When plasma glucose levels rise postprandially, facilitated transport by the low affinity glucose transporter GLUT2 (*K*_m_ = 15–20 mM), the primary glucose transporter present on β-cells (56), is initiated (Figure 1). In β-cells, glucose is metabolized through the TCA cycle to yield ATP, the main stimulator of insulin secretion (37). ATP acts by closing potassium channels $\left(K_{\mathrm{ATP}}^{+}\right)$ which leads to depolarization of the β-cell (37). Exocytosis of insulin containing SGs occurs when Ca^2+^ levels rise after opening of voltage-gated Ca^2+^-channels (37, 38).

Many amplifying signals modify islet secretion (57). There is support for amino acids influencing the endocrine function of the pancreas (4–6), and in particular glutamate, GABA, and glutamine are postulated to play a role in the regulation of hormone secretion (4, 7). Deletion of glutamate dehydrogenase (GDH), which catalyzes the conversion between glutamate and the TCA cycle intermediate α-ketoglutarate, decreases glucose-induced insulin secretion by approximately 40% (58). In addition, glutamine’s ability to evoke insulin release is decreased in isolated islets from GDH knockouts (59). Furthermore, the very close proximity between the different cell types in human islets facilitates flux of substrates and paracrine signaling for the orchestration of islet secretion (Figure 1) (60, 61). Thus, there are compelling evidence for glutamate, GABA, and glutamine being involved in regulating islet hormone secretion.

## Amino Acid Transporters in Islet Secretion

As described above, glutamate and GABA, their specific receptors and effects, as well as the enzymes involved in their metabolism have all been shown in the islet. However, the mechanisms involved in the transport of amino acids across plasma and vesicle membranes and mode of action in the islet cells are poorly understood. Interestingly, recent advances in the characterization of amino acid transporters in the islets reveal peculiar insight into novel mechanisms for signaling in the islets.

### Vesicular glutamate and GABA transporters are localized on SLMVs as well as SGs in islet cells

Glutamate is transported into synaptic vesicles by the vesicular glutamate transporters (VGLUT1-3) of the Slc17 family (62), whereas VGAT (Slc32) transports the inhibitory neurotransmitters GABA and glycine (63, 64). In the CNS, the VGLUTs and VGAT may be localized on different subsets of synaptic vesicles or co-localized on the same vesicles (65–68). This differential localization of vesicular transporters allows for co-release or differential release of transmitters, as well as concerted action to increase packaging.

Islet cells express both VGLUTs and VGAT (Figure 1). In α-cells, VGLUT1 and VGLUT2 are found on glucagon containing SGs (43, 69), supporting the finding that there is co-release of glucagon and glutamate from α-cells (42). On the SLMVs of the α-cells, there is expression of the 57-kDa isoform of VGAT found in the CNS (55, 70–72), but in addition a novel 52.5 kDa isoform is found on the SGs (72). The expression levels of the 52.5-kDa VGAT isoform increase with increasing glucose levels, opposite to the regulation of the transcription and activity of VGLUT1 and VGLUT2 which is down-regulated by increasing glucose levels in islet cell cultures (73). Thus, granular loading of glutamate, GABA, and glycine changes with changing glycemic conditions, allowing for fine-tuning of hormone secretion.

In the β-cells, VGLUT3 is found on both SGs and SLMVs (Figure 1A) (43). However, the SGs contain little glutamate (43), and direct stimulation of granule secretion with sulfonylurea tolbutamide, which closes ATP-sensitive potassium channels, does not lead to glutamate release from β-cells (74), questioning the role of SG release of glutamate as important for signaling. Unlike β-cell SGs, SLMVs accumulate glutamate and express high levels of VGLUT3 (43). It has been hypothesized that the main function of SLMV exocytosis is to supply the plasma membrane with the proteins and lipids necessary to withstand the stress from substances like Zn^2+^ secreted together with insulin (75). However, the presence of VGLUT3 on and glutamate in SLMVs might indicate that glutamate release from β-cells participates in feedback mechanisms regulating insulin secretion. This is supported by the fact that exocytosis of SLMVs and SGs is differentially regulated through two cAMP-dependent pathways (75). β-cells also express the 57-kDa isoform of VGAT on SLMVs (55, 71, 72), which probably primarily supports GABA release, as β-cells contain much higher levels of GABA compared to glycine (55).

Thus, both islet SGs and SLMVs have the machinery necessary to load them with glutamate and/or GABA and glycine, which can then be released in an exocytotic manner.

### Plasma membrane glutamate transporters may terminate fast transmission as well as contribute to a novel mechanism for the maturation of SGs

Na^+^-dependent glutamate transport across the cell membrane in islet cells (76), probably facilitated by the glutamate transporter GLT/EAAT2 (Slc1) expressed on the plasma membrane of pancreatic β-cells (77), can terminate glutamate signaling (Figure 1A). In fact, glutamate transport by EAAT2 prevents glutamate induced excitotoxicity as down-regulation of EAAT2 lead to increased β-cell death, whereas up-regulation increased β-cell survival (77).

EAAT2 has also been shown to be strongly expressed on the membrane of SGs in β-cells (43), and co-localizes with both chromogranin, a neuroendocrine secretory protein in granules, and insulin in β-cells (77). As discussed above, the SGs also express VGLUT3, but there is very little concomitant release of glutamate and insulin (74) and glutamate levels inside SGs are insignificant (43). The presence of both of these transporters in the β-cells raises the possibility of alternative mechanisms through which glutamate can act as an intracellular signaling molecule modulating the release of insulin.

Gammelsæter and co-workers postulated that glutamate transport might participate in the maturation of insulin in the SGs, and thus the regulation of insulin release (43). SGs contain a vesicular H^+^-ATPase which pumps protons into the SGs (Figure 1B). Uptake of negatively charged glutamate by VGLUT3 sets up a counter-charge movement which decreases the granular membrane polarization allowing sustained transport by the H^+^-ATPase. If glutamate leaves the SGs through EAAT2, three positively charged sodium ions and a proton will be exported, while one potassium ion will enter the SG. Thus, there will be a net export of positive charge, further counteracting inhibition of the H^+^-ATPase, allowing for further acidification of the SG. Acidification is crucial for the conversion of pro-insulin to active insulin (78, 79), and it is thought to be important for the exocytotic process (79). Interestingly, perturbation of the granular acidification by genetic deletion of the chloride channel ClC-3, which resides on the SGs (Figure 1B), also results in abolished insulin release (80). Chloride ions also regulate or penetrate VGLUTs and VGAT (81, 82), and may acidify SG in multiple ways. Altogether, these data suggest that VGLUT3 probably works in consortium with EAAT2 to acidify the SGs through glutamate import and subsequent export, which further regulates the level of insulin released upon glucose stimulation.

### Members of the Slc38 family of amino acid transporters might both supply glutamine for the formation of glutamate and GABA and contribute to plasma membrane depolarization in the islets of Langerhans

**Figure 2:**
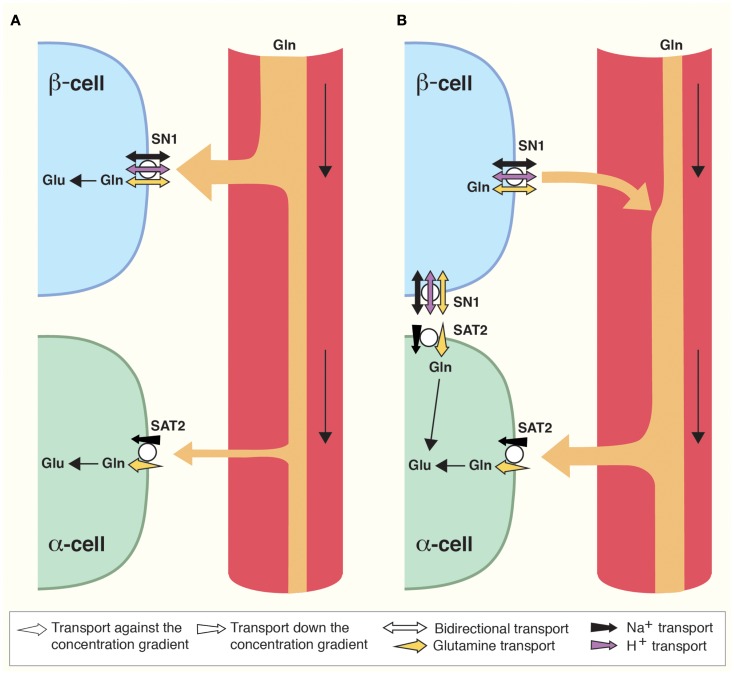
**Direction of glutamine transport in the islet cells of Langerhans is differential and dependent on prandial status**. **(A)** Postprandially, blood supplied to the islets is enriched in glucose and amino acids such as glutamine (Gln). Gln is captured by SN1 and metabolized in β-cells to stimulate insulin secretion as described (Figure 1). As a result, blood glucose levels are reduced. **(B)** Interprandially, blood supplied to the islets has lower concentrations of Gln and glucose. Gln now preferentially activates SAT2 on α-cells due to its higher affinity for Gln. In addition, changes in the Gln gradient across the cell membrane of β-cells may stimulate SN1 to release glutamine which can then be taken up by adjacent α-cells. Enhanced Gln metabolism in α-cells stimulates secretion of glucagon which raises blood glucose levels.

The islets of Langerhans express transcripts for several members of the Slc38 gene family (Figures 1A and 2) (83). Immunofluorescence co-labeling with antibodies against SN1 and SAT2 with specific markers for the different cell populations of the islets showed SN1 at the plasma membrane of β-cells, whereas SAT2 was found on the membrane of α-cells. This distribution resembles the complementary expression pattern of SAT2 and SN1 in the CNS, where SN1 and SAT2 transport glutamine from astrocytes to glutamatergic neurons for the replenishment of glutamate (23).

SN1 translocates glutamine coupled to a symport of Na^+^ (18). In addition, it is sensitive to pH, and SN1 both transports protons coupled to the transport of glutamine and allows for flux of protons not stoichiometrically dependent on the movement of substrate (21, 84, 85). Under most physiological conditions, the electroneutral SN1 may therefore transport glutamine bidirectionally (18, 21, 84–87). SN1 at the membrane of β-cells might therefore accumulate glutamine for glutamate and GABA synthesis (Figures 1A and 2), but at different feeding status it may contribute to the release of glutamine (Figure 2), as discussed below.

Glutamine has been shown to have a profound effect on insulin secretion (4–6). Increased levels of glutamate and GABA in β-cells from replenishment by glutamine are not sufficient to explain this strong effect on secretion. It is therefore likely that glutamine contributes to the regulation of insulin secretion through additional mechanisms. First, glutamine taken up by SN1 might stimulate insulin secretion through the conversion of glutamine to glutamate by PAG (Figure 1A), which is present in β-cells (53). This is in agreement with earlier findings showing conversion of glutamine to glutamate in islet cells (88, 89). In the β-cell, glutamate can then enter the TCA cycle through the action of GDH, and stimulate insulin secretion in the same $K_{\mathrm{ATP}}^{+}$-dependent way as glucose (53). This is supported by the fact that, in isolated islets from GDH knockouts there is an abrogation of insulin release evoked by glutamine (59). Alternatively, glutamate flux through VGLUT3 and EAAT2 on SGs might contribute to the maturation of insulin, as described above (Figure 1B). Glutamine transport by SN1 can also contribute to the depolarization of the membrane of β-cells. As already mentioned SN1 is sodium dependent and exhibits a channel-like activity, for e.g., protons, inducing inward currents (18, 21, 85). Due to these characteristics, SN1 can have a depolarizing effect upon transport of glutamine (21). The potassium channel $K_{\mathrm{ATP}}^{+}$ is responsible for the depolarization initiated insulin release in response to glucose. In knockout mice with a loss of function mutation of the $K_{\mathrm{ATP}}^{+}$, insulin secretion can be triggered by amino acids (4). This effect decreased 60% if glutamine was omitted, which was suggested to be due to the depolarizing effect of glutamine transport into the cell (4), and the conveyer may well be SN1 (83). Thus, glutamine uptake by SN1 due to elevated serum levels of glutamine may not only stimulate insulin secretion through conversion to glutamate, but also by amplifying the depolarization of the plasma membrane.

Lack of coupling to proton translocation enables the SATs to utilize both the electrical and the chemical gradients of sodium, creating higher glutamine concentration gradients compared to SN1 (19, 23, 90). In addition, SAT2 has higher affinity for glutamine than SN1 (90). According to our findings, SAT2 is expressed on α-cells (Figures 1A and 2) (83). In α-cells, glutamate is exocytosed together with glucagon when blood glucose levels are low (42). As SAT2 is able to create a large concentration gradient of glutamine across the membrane, SAT2 might therefore ensure a constant supply of glutamine for the production of glutamate. Interestingly, when the interprandial serum levels of glutamine are low, flux reversal of SN1 might assure substrate for transport by SAT2 (Figure 2). Since system A transport depolarizes cells (19, 90), its activity might also stimulate glucagon secretion in a similar manner to the postulated role of SN1 in β-cell depolarization.

All in all, as SN1 changes direction of transport at glutamine concentrations well within physiological fluctuations in amino acid content of the blood (18), SN1 might be a convenient conveyer of plasma amino acid level stimuli for islet cell secretion. We have therefore proposed that SN1 works as a sensor of nutritional status (83). When the blood glutamine concentration is high it may mediate uptake of glutamine in β-cells. However, when blood glutamine concentration decreases between meals it may mediate release from β-cells. This will promote the ability of SAT2 to import glutamine into α-cells and thus stimulate glucagon secretion (Figure 2). Furthermore, insulin has been shown to regulate SN1 expression and function (28), allowing for possible additional feedback mechanisms.

## Conclusion

The main components of synaptic transmission are present in the islet cells implicating similarities in signaling pathways. This notion is further reinforced by the fact that human neural progenitor cells may be differentiated to produce insulin (1). In the CNS, synaptic transmission is based on a tripartite synapse where the perisynaptic astroglial cells actively participate in fine-tuning and termination of the signal and in supplying precursors for the neurotransmitters. An analog to the tripartite synapse does not exist in the islets of Langerhans. However, secretion by the different islet cells is regulated by both paracrine and autocrine signaling, which again depends on glutamate and GABA. Moreover, the amino acid transporters involved in the GGG cycle in the CNS do have the corresponding and necessary localizations to support glutamine-mediated synthesis of glutamate and GABA, their exocytotic release and termination of the signal in the islets of Langerhans. Depolarization of the plasma membrane due to inward transport by SN1 upon elevated plasma glutamine concentration and/or its metabolism to glutamate and ATP might be important regulators of insulin secretion after protein rich meals. Similarly, glutamine from plasma or from β-cells upon reversed action of SN1 may activate SAT2 on α-cells (Figure 2). Such glutamine transport may depolarize α-cells and/or glutamine may be metabolized to glutamate to regulate glucagon secretion. This resembles the situation in the CNS where glutamine uptake through SAT1 depolarizes the nerve terminal and regulate transmitter release (19). The co-localization of EAAT2 and VGLUT3 on insulin containing SGs supports a cell-specific flux of glutamate through the granules, which may facilitate the acidification necessary for insulin maturation prior to secretion. Thus, there are several striking similarities, but there are also important differences in the functional roles of the amino acid transporters in these two tissues. Altogether, our data suggest that in addition to insulin secretion in the islets of Langerhans and the brain-centered glucoregulatory system (3), amino acid metabolism and amino acid transporters might also be potential therapeutic targets in diabetes.

## Author Contributions

This review was written by Monica Jenstad and Farrukh Abbas Chaudhry in collaboration.

## Conflict of Interest Statement

The authors declare that the research was conducted in the absence of any commercial or financial relationships that could be construed as a potential conflict of interest.
